# Barriers to Mammography Screening among Black Women at a Community Health Center in South Florida, USA

**DOI:** 10.18103/mra.v11i4.3814

**Published:** 2023-04-25

**Authors:** Tarsha Jones, Karen Wisdom-Chambers, Katherine Freeman, Karethy Edwards

**Affiliations:** Professor John F. Wymer, Jr. Endowed Distinguished Professor CEO FAU/NCHA Community Health Center. Member, National Advisory Committee, American Nurses Association/Substance Abuse and Mental Health Services Administration Minority Fellowship Program, Florida Atlantic University

## Abstract

**Background:**

In the United States (US), Black/African American women suffer disproportionately from breast cancer health disparities with a 40% higher death rate compared to White women. Mammography screening is considered a critical tool in mitigating disparities, yet Black women experience barriers to screening and are more likely to be diagnosed with advanced-stage breast cancer. The purpose of this study was to assess the relative frequency of mammography screening and to examine perceived and actual barriers to screening among women who receive care in our nurse-led community health center.

**Methods:**

We conducted a survey examining frequency of mammography screening and beliefs about breast cancer including perceived susceptibility, perceived benefits, and perceived barriers to mammography screening, guided by the Champion Health Belief Model.

**Results:**

A total of 30 Black/African American women completed the survey. The mean age of the participants was 54.3 years ± 9.17 (SD); 43.3% had a high school education or less; 50% had incomes below $60,000 per year; 26.7% were uninsured; 10% were on Medicaid; and only 50% were working full-time. We found that only half of the participants reported having annual mammograms 16 (53.3%), 1 (3.3%) every 6 months, 8 (26.6%) every 2–3 years, and 5 (16.7%) never had a mammogram in their lifetime. Frequently cited barriers included: ‘getting a mammogram would be inconvenient for me’; ‘getting a mammogram could cause breast cancer’; ‘the treatment I would get for breast cancer would be worse than the cancer itself’; ‘being treated for breast cancer would cause me a lot of problems’; ‘other health problems would keep me from having a mammogram’; concern about pain with having a mammogram would keep me from having one; and not being able to afford a mammogram would keep me from having one’. Having no health insurance was also a barrier.

**Conclusion:**

This study found suboptimal utilization of annual screening mammograms among low-income Black women at a community health center in Florida and women reported several barriers. Given the high mortality rate of breast cancer among Black/African American women, we have integrated a Patient Navigator in our health system to reduce barriers to breast cancer screening, follow-up care, and to facilitate timely access to treatment, thus ultimately reducing breast cancer health disparities and promoting health equity.

## Introduction:

In the United States (US), breast cancer is a major public health issue that is compounded by racial and ethnic health disparities.^1,2^ Black women represent a vulnerable population^3^ and public health experts and researchers have argued that evidenced-based targeted interventions that promote equity and improve access to timely screening mammography, early detection, and quality breast cancer treatment, could improve survival outcomes for this population.^2,4^ An estimated 36,260 new cases of breast cancer in the United States (US) were expected to occur among Black women in 2022, and it is estimated that approximately 6,800 Black women were expected to die from breast cancer. ^5^ Although Black women in the US have a lower incidence of breast cancer compared to White women (127.8 vs. 133.7 per 100,000),^5^ the unacceptably high racial disparity in breast cancer mortality persists, with a 40% higher death rate in Black women (27.6 deaths per 100,000 in 2016–2020) compared to White women (19.7 deaths per 100,000).^5,6^ Furthermore, Black women diagnosed before age 50 have twice the death rate from breast cancer compared to White women under 50 (12.1 vs. 6.5 deaths per 100,000).^5^ Strikingly, breast cancer is the leading cause of cancer-related deaths for Black women, further underscoring the need to address this public health challenge.^5^

When examining racial/ethnic cancer health disparities, it is important to note that Black women are largely impacted by social determinants of health (SDOH),^7^ with poverty being a critical driver of breast cancer health disparities.^1^ Black women represent approximately 22.3% of all women living in poverty^8^ in the US, they experience higher unemployment rates compared to White women, are more likely to be the head of households supporting multiple dependents, and are more likely to live in underserved neighborhoods with public housing developments that are racially segregated.^7,9^ Black women are also more often uninsured and lack access to quality healthcare services, making it very difficult for them to access and sustain recommended mammography screening. Access to care is an important issue, as Black women have high rates of chronic conditions such as cardiovascular disease, diabetes, obesity and other comorbidities that require consistent reliable access to care.^7^ Furthermore, when Black women are diagnosed with breast cancer, tumors are biologically aggressive with unfavorable characteristics, and later stage at diagnosis.^7^ Among Black/African-American women, 20% of breast cancers are triple negative breast (TNBC), an aggressive subtype of breast cancer with limited treatment options, higher likelihood of metastasis and recurrence.^6,9^ Furthermore, Black women are impacted by breast cancer susceptibility genes, most notably *BRCA1* and *BRCA2* pathogenic variants or gene mutations and should undergo genetic testing to determine their hereditary cancer risk.^10–13^ Late-stage diagnosis of breast cancer is also a challenge for Black women because the tumor has already progressed to an advanced stage (III & IV), which reduces the treatment options and reduces the likelihood of survival; thus early mammography screening is essential. It is known that factors contributing to breast cancer health disparities are multifactorial and include an interplay of personal, provider, and system-level factors.^13^

### Mammography

Mammography screening is associated with 20% to 25% reduction in breast cancer mortality.^14–16^ Previous studies have found that women who have regular mammograms are likely to detect breast cancer at an early stage, are less likely to need to remove the entire breast through a mastectomy, or aggressive forms of breast cancer treatment.^17^

The U.S Preventive Services Task Force (USPSTF), gold standard for screening, recommends that women ages 50 to 74 years old receive a mammogram every 2 years and younger women ages 40–49 years old should make an informed decision about screening with their health care providers.^18,19^ However, the American College of Radiology (ACR) and the Society of Breast Imaging (SBI) recommends that average risk women start having mammograms at age 40.^20^ This is especially important for young Black women to have conversations with their health care providers about their family history of breast cancer and to discuss their personal cancer risk rather than wait until age 50 to have their first mammogram.

Healthy People (HP) 2030, our nation’s health promotion plan, has a target goal to increase the proportion of women who get screened for breast cancer from 76.4% to 80.5%.^21^ HP 2030’s overarching goals are to eliminate health disparities, achieve health equity, and to improve health outcomes for all. Black women are more likely to be screened for mammography at non-accredited facilities located at safety-net hospitals in minority serving communities.^22,23^ Furthermore, when an abnormal result is found on a mammogram, Black women experience delays in follow-up, diagnosis, and treatment.^24^ Documented barriers to mammography screening among Black women encompass individual, structural, and systems-level barriers and contribute to a higher mortality rate among this population.^1,23,25–29^ Increasing mammography screening rates among Black women in underserved communities is responsive to the HP 2030 goals.

Federally qualified health centers (FQHC) are well positioned to mitigate barriers to breast cancer screening among vulnerable populations, as they are accessible within underserved communities and serve as a regular source of care with trusted healthcare providers. The setting for our study is the Florida Atlantic University (FAU)/Northwest Community Health Alliance Community Health Center (CHC), a FQHC Look -A Like operated by the Christine E. Lynn College of Nursing (CON). The FAU CON opened its first Nurse-led Community Health Center in West Palm Beach, Florida in 2014 to increase access to healthcare services for residents in the West Gate neighborhood of West Palm Beach and a second site was opened in 2020, to expand services and to meet the growing needs of the underserved populations within the community. Our CHC is the first in Florida to be designated by the United States Health Resources and Services Administration (HRSA), as a “Federally Qualified Health Center (FQHC) Look-Alike.” This is important as our CHC is able to improve access to quality screenings, healthcare, and treatments for vulnerable underserved populations.

The geographic setting of this study is also important, as 17% of the population in Florida are Black or African-American alone, compared to 13.6% of the US population.^30^ Furthermore, Florida is home to the second largest Black immigrant population, coming primarily from Caribbean countries such as Haiti and Jamaica. Given the health disparities in breast cancer outcomes as well as the cultural diversity within the Black community, there remains a need to study Black women.

The purpose of this study was to assess the relative frequency of mammography screening and to examine perceived and actual barriers to mammography screening encountered by a sample of Black women who receive care at our CHC. We also reviewed medical records to determine the documented mammography screening rates of minority women at our CHC who are primarily Black and Hispanic women.

## Methods:

### Theoretical Framework

This study was guided by the Health Belief Model (HBM), a widely used conceptual framework developed in 1950’s by psychologists in the Public Health Service to explain people’s failure to participate in programs to prevent and detect disease and to improve their health behaviors.^31^ The model has been expanded over the years and our study is guided by Dr. Victoria Champion’s Health Belief Model Scale (CHBMS), an instrument that is guided by the HBM constructs.^32^ The revised Champion’s Health Belief Model posits that health behaviors and screening behaviors are influenced by health beliefs such as perceived susceptibility, perceived seriousness, perceived benefits, perceived barriers to action, self-efficacy, and health motivation.^31,33^ This scale was developed by Dr. Champion in 1984, revised in 1993, 1997 and finalized in 1999 to focus on health beliefs about mammography screening.^31^ For this study, we used three subscales from the CHBMS: *perceived susceptibility* [the belief about the probability or likelihood of getting a disease or condition]; *perceived benefits* [the beliefs about the benefits of the available actions for reducing the threat]; and *perceived barriers* [the beliefs about the potential negative aspects of a particular health action].^31,34^

### Study Design and Context

This survey study was conducted from February to August 2021 at the FAU CHC in South Florida. Participants were recruited from the FAU/NCHA Community Health Center service area located in West Palm Beach, a city in Palm Beach County, where the majority of the population is Black [indigenous of the African diaspora such as African-Americans, Haitian, Jamaicans and other Caribbean descent]; Hispanic [Black or White Hispanics]. About 47 percent of the population in West Palm Beach live below 200 percent of the poverty-level. More than 20 percent of the population in West Palm Beach are uninsured, compared to 15 percent in Palm Beach County and Florida overall. The entire service area of the FAU CHC is designated as a primary care, dental and mental health “Health Professional Shortage Area.” People in poverty often lack health literacy and access to resources needed to develop sustained care partnerships essential for their long-term health and well-being. FAU’s Community Health Centers are strategically located in these medically underserved areas to provide comprehensive and culturally sensitive primary and psychiatric care services to any individual who walks through the doors regardless of their ability to pay. The College’s Nurse Practitioners (NP), registered nurses, and other health care providers staff the CHC. The CHC is grounded in the College’s Caring Science philosophy and serves as the training site for current and future nurses and nurse scientists.

### Medical Record Review

We conducted a retrospective chart review of all racial and ethnic minority patients, majority of whom are Black and Hispanic women, of mammography screening age in the electronic health record (EHR) system at the CHC. From the medical record review, we found that a total of 392 underserved women, both English and Spanish speakers, between the ages of 40–74 years old were eligible for an annual screening mammogram. Only 31% (123/392) had a documented mammogram in the EHR within the past 2 years (2019–2021). Among the 392 women, 245 (62.5%) identified as Black/African-American. Several organizations recommend mammography screening beginning at age 40, and providers at our CHC use these guidelines when identifying and referring women for mammograms.^35^ EHR documentation showed that some of these women had not received care at the CHC since 2016 and may have been lost to follow-up, whereas others were given the script to have a mammogram by a healthcare provider but did not receive mammograms due to possible system-level barriers. At the time of the medical record review, women who could not afford to have a mammogram could have received support under the Florida Breast and Cervical Cancer Early Detection Program, which offers free or low-cost screenings for women who meet eligibility criteria; however, women may have encountered delays in getting into a mammogram facility due to the limited access to free mammograms sites or personal barriers they encountered. Some patients with mammogram scripts did not receive one if they were uninsured, demonstrating a critical need for intervention.

### Participants

Participants for this study were identified from the medical record review described above. Eligible participants included women who identified as Black or African American, spoke English as a primary language and could read and write in English, between the ages of 40–74 years, and had an address or contact phone number in the medical record. We mailed invitation letters to a convenient sample of English-speaking participants on the medical record review list, with addresses in the medical record and made follow-up phone calls to assess their interest in this study and to address any further questions they had. As this was a small pilot study to gain insight into the needs of this population, we stopped recruitment at 30 women. Participants received a $15 gift card after completing the survey.

### Survey

The survey was conducted from February 2021 to August 2021 at the FAU CHC in South Florida. It consisted of 48 items encompassing demographic questions, such as age, race, ethnicity, country of birth, language spoken at home, education, income, insurance, and employment status. Clinical and behavioral questions included: have you been diagnosed with breast cancer, what treatments did you receive, family history of breast cancer, how often do you have a mammogram, when was your last mammogram, how often do you conduct a breast self-exam (BSE) an open ended-item asked participant if they had not had a mammogram, please explain why. The survey was administered by a doctorate of nursing practice (DNP) student. Most women completed the survey over the telephone and some during a scheduled clinic visit. The entire survey took approximately 30 minutes to complete.

The second part of the survey included three subscales from the CHBMS. Perceived Susceptibility (4 items), Perceived Benefit (4 items), and Perceived Barriers (19 items) to Breast Cancer Screening subscales. Participants were asked to rate each item on the subscales using a 5-point Likert Scale: 1-Very Unlikely; 2- Somewhat Unlikely; 3- Neutral; 4- Somewhat Likely; and 5- Very Likely. The highest score on each item is a 5- “Very Likely”, which indicates positive beliefs about breast cancer screening, with the exception of the barriers scale where higher scores indicates greater perceived barriers.

### Ethics Statement

The study was approved by the Florida Atlantic University Institutional Review Board (IRB) and all participants gave verbal informed consent before they enrolled in the study. The researcher explained to participants that participation is entirely voluntary, the data will be deidentified to promote confidentiality, and that results will be presented in aggregate and will be used for research purposes only.

### Data Analysis

Relative frequencies are presented as descriptive statistics for categorical and short-scale ordinal variables, and means and standard deviations for normally distributed continuous variables. Analyses were performed using SAS Software Package Version 9.4 (SAS Institute, Cary, NC).

## Results

Baseline demographic characteristics of the 30 participants are presented in Table 1. The mean age was 54.3 ± 9.17 years (minimum 40- maximum 68). All women identified as Black and one woman was of Hispanic ethnicity. Of the participants, 43.3% had a high school education or less; 50% had incomes below $60,000 per year; 26.7% were uninsured; 10% were on Medicaid; and only 50% were working full-time. In terms of country of birth, 12 (40%) women were US-born, while the remainder of the women were born in Jamaica, Bahamas, Barbados, Brazil, and Haiti. The majority of the women reported having an annual mammogram (n=16, 53.3%); 1 (3.3%) woman reported having a mammogram every 6 months; 4 (13.3%) had mammograms every 2 years; for 2 (6.7%) of the women, it had been more than 3 years since they last had a mammogram; 2 (6.7%) of the women couldn’t remember when they last had a mammogram and said they get it when they remember; and 5 (16.7%) reported that they never had a mammogram. Eight women (47.0%) reported that they perform monthly breast self-exam and 9 (30%) women had a family history of breast cancer.

### Perceived risk

We found that most participants 20 (67%) had a low perceived risk of breast cancer, and reported that it is ‘very unlikely’ that they will get breast cancer in the next five years. Most participants 17 (57%) also stated that it is ‘very unlikely’ that they will get breast cancer in the next 10 years, and, most of the participants 18 (60%) believed that it is ‘very unlikely’ that they will get breast cancer in their lifetime.

### Perceived Benefits

We also found that most participants viewed mammography as beneficial, as 24 (80%) of them believed that if breast cancer is found early, it’s likely that the cancer can be treated successfully. Most participants (90%) indicated that having a mammogram could help find breast cancer when it’s first getting started; 28 (93%) believed that having mammogram could help find a breast lump before felt; and 21 (70%) believed that having a mammogram will decrease their chances of dying from breast cancer.

### Perceived Barriers

Perceived barriers to mammography screening are shown in Table 2. Top cited barriers included the following: 7 women reported that it’s very likely that ‘getting a mammogram would be inconvenient for me’; 3 women felt that it is very likely that ‘getting a mammogram could cause breast cancer’ and 9 women were neutral; 5 women reported that it is very likely that ‘the treatment I would get for breast cancer would be worse than the cancer itself’, 1 woman felt this is somewhat likely and 10 women were neutral; 11 women reported that it is very likely that ‘being treated for breast cancer would cause me a lot of problems’, 2 somewhat likely, and 4 were neutral; 7 women felt that that ‘other health problems would keep me from having a mammogram’; 10 women felt that ‘not being able to afford a mammogram would keep me from having one’ 3 women felt that ‘being treated rudely at the mammogram centers would keep me from having a mammogram’; and 5 women reported that ‘concern about pain with having a mammogram would keep me from having one.’

In one open-ended item in the survey that asked, *“if you have not received a mammogram, please explain why?”* The most cited barriers to not having a mammogram include: (1) No insurance. One participant stated, “I am waiting to get insurance because if something is found before that time, I might not be able to get insurance.” (2) COVID-19 caused some restrictions to access. (3) Mitigating Radiation Risk: Another participant stated, “I do mammograms at 3 years because I don’t want too much exposure.” (4) Lack of Knowledge: one participant stated, “I was not at the required age before but I will get it now that I’m scheduled. I’m motivated because I want to stay in good health.”

## Discussion

Overall, a little over half of the women in our study reported having annual mammograms, the remaining women reported having mammograms every 2–3 years, and some women never had a mammogram in their lifetime, despite being age 40 years old. According to the American College of Radiology (ACR), one in six women are diagnosed with breast cancer are in their 40’s.^36^ ACR and the Society of Breast Imaging (SBI) recommends that average risk women start having mammograms at age 40 to reduce the likelihood of extensive treatments and breast cancer.^20^ Black women are particularly vulnerable because they have a higher incidence of early onset breast cancer and those diagnosed before age 50, have twice the death rate from breast cancer compared to White women^37^. While the United States Preventive Services Task Force (USPSTF) recommend screening mammograms beginning at age 50 for average-risk women;^35,38^ these guidelines may be putting Black women at a disadvantage as they are more likely to be diagnosed with breast cancer at younger age with advanced stage breast cancer, and with a 40% higher mortality rate compared to White women.^5,39^ In fact, a recent study found that 23% of breast cancers in Black women occur before age 50, thus making it essential for healthcare providers to educate younger Black women about breast cancer, assess their risk, and to facilitate timely and consistent practice of annual mammograms.^28^ Prior studies have found lower screening mammography rates among racial and ethnic minority women and underserved lower-income Black women.^19,29^

### Perceived Risks

In our study, we found that most Black women had a low breast cancer risk perception; 67% of them reported that it is ‘very unlikely’ that they will get breast cancer in the next 5 years and (60%) of the them believed that it is ‘very unlikely’ that they will get breast cancer in their lifetime. Prior studies also show that perceived risk is often inaccurate among Black or African-America women, as they tend to underestimate their risk, despite having higher mortality rates from breast cancer compared to other racial/ethnic groups.^19,40^ Our study provides insights into the health beliefs of Black women and demonstrates that there is an opportunity to increase education and awareness about objective breast cancer risk and to promote accurate risk perception among Black women, particularly those with a family history of breast cancer. In our study, 30% of women reported that they have a family history of breast cancer and one woman in our sample shared that she was diagnosed with breast cancer. Research shows that inaccurate perceptions of risk negatively impact breast cancer screening and must be addressed to promote optimal breast cancer screening.^41^ The literature also shows the impact of spiritualty and religion in the Black community and the role of one’s faith on their decision-making about health. Some Black women may not worry about breast cancer and say “it is in God’s hand”,^42^ thus they are not as proactive about their breast health concerns. This speaks to the importance of healthcare providers, engaging in shared-decision making and communicating risk with vulnerable populations such as Black women. It is imperative that providers, including nurse practitioners, utilize breast cancer risk assessment tools to determine objective breast cancer risk and to educate Black women about their absolute and relative risk of developing breast cancer and when to begin breast cancer screening.^43^ Previous studies have found that while healthcare providers spend time with women discussing mammography screening recommendations, they tend to spend less time discussing the woman’s risk and modifiable risk behaviors.^44,45^

### Perceived Benefits

Interestingly, we found that the participants perceived mammograms as very beneficial. Majority, (80%) of the women, believed that ‘if breast cancer is found early, it’s likely that the cancer can be successfully treated’ and 90% indicated that ‘having a mammogram could help find breast cancer when it is first getting started.’ These findings suggest that Black women actually see the benefits of having an annual mammogram but some perceived barriers may be preventing them from acting upon their desire to have a mammogram due to lack of awareness about the resources available to support uninsured or under-insured women. As shown in Table 3, in Palm Beach County, Florida, federally funded programs and numerous non-profit organizations such as Promise Fund of Florida, FoundCare, and Genesis provide free and discounted state-of-the art 3D mammograms for women who cannot afford them because they are uninsured, under-insured, or have limited financial resources. Having access to these facilities in one’s neighborhood is important as previous studies have found that neighborhoods lacking quality advanced imaging facilities drive inequitable breast cancer outcomes, particularly among racial/ethnic minority women.^46,47^

### Perceived Barriers

While there are notable benefits to mammography screening, we found that many Black women perceive barriers to mammography screening and these beliefs should be taken into consideration when designing interventions to increase breast cancer screening. It appears from the top barriers reported in the survey, mammography screening was perceived as inconvenient for some women, possibly due to competing demands of childcare responsibilities, difficulty with transportation, or taking time off from work to have a mammogram. Some women reported that they believe that a mammogram ‘could cause breast cancer’ and others were concerned about being ‘exposed to radiation’ during their mammograms, indicating that the risks of having a mammogram were of concern. Others felt that treatment for breast cancer would cause ‘a lot of problems’ and would be worse than the cancer itself, indicating a need for education regarding effective breast cancer treatment options, particularly when breast cancers are detected at an early stage. The COVID-19 pandemic was also a critical barrier that delayed mammography screening for these women. Furthermore, some women felt that other health problems they were experiencing (co-morbidities) would keep them from having a mammogram, which is not surprising given that Black women have a high prevalence of chronic conditions.^7^ A few women felt that ‘being treated rudely’ at mammogram facilities would keep them from getting a mammogram in the future. There is a body of literature that demonstrates that Black or African-American women, particularly those of low-income, have personal fears and concerns about mammography screening and tend to have a fatalist belief that breast cancer will have a devasting impact on their lives.^42,48–53^ Collectively, these findings demonstrate the need for culturally targeted interventions to address the concerns of Black women in order to reduce barriers to mammography screening.

### Addressing Social Determinants of Health

Notably, several women felt that not being able to afford a mammogram would keep them from getting one, which is an important barrier to address since Black women are largely impacted by social determinants of health. Some women also stated that having no health insurance was a barrier to screening for them. In our sample, half of the women (50%) who answered the income question had annual incomes below $60,000. Living in poverty is a substantial risk factor for poor health outcomes because women who are poor do not have discretionary incomes to use as co-pays for healthcare services. This demonstrates the importance of providing these women with free life-saving mammograms. Additionally, 8 of the women (26.7%) were uninsured at the time of the survey, 5 (16.7%) were unemployed, and 4 (13.3%) were disabled, indicating the vulnerability of this population. It is also important to note that 37% of the participants in our study were Haitian immigrants, several of whom expressed that they felt more comfortable communicating in Haitian Creole rather than in English. Our nurse-led FAU/NCHA Community Health Center provides patient-centered care, culturally, and linguistically appropriate care to Black women and other underserved populations. At our CHC, providers speak Spanish and Haitian Creole to better understand and meet the needs of the population.

### Practice Implications

Our findings clearly indicate a need for a Patient Navigator (PN) in our community health center (CHC) to address the unique needs of the population. PN is an evidenced-based intervention that is effective in increasing mammography screening rates in underserved women. Since this study was completed, our CHC integrated a PN as a member of the healthcare team. Most women who receive care at our CHC are 200% below the federal poverty level, uninsured, unemployed, and many are homeless. Previous studies have found that PN significantly reduces delays in breast cancer screening, improves the likelihood of follow-up after abnormal screening and access to timely breast cancer treatment; overall, PN has a positive effect on the lives of women and could substantially reduce breast cancer health disparities.^54–58^ The PN provides support across the continuum of care and addresses perceived and actual barriers to healthcare and mammography screening. The PN addresses the concerns of women who need to be seen by a provider for breast symptoms or pain and makes referrals for any abnormal or suspicious findings. Most importantly, the PN tracks and ensures that all women are receiving timely annual mammograms. When women are diagnosed with breast cancer, the PN determines the best facility for women to begin timely treatment and assists them with necessary follow-up care. The PN at our health center also connect women to social services for financial and transportation assistance, participate in community outreach, and conduct culturally appropriate breast health education within underserved communities in Florida. A subsequent article will report the outcomes of the Patient Navigation Program at our CHC, as PN represents an important evidence-based intervention to mitigate health disparities.

## Study Limitations

Several limitations of our study warrant discussion. First, this study was conducted at a single community health center targeting a sample of Black women, which may limit the generalizability of our findings to other populations and geographic areas. Second, the sample size was small, which precludes hypothesis testing and multivariate analysis. Nonetheless, it is important to document the prevalence of barriers that are unique to underserved populations in order to understand the needs of the population and to address these barriers. Future work will include a larger diverse sample of women who receive Patient Navigation.

## Conclusion

This study found suboptimal utilization of annual screening mammograms for Black/African women at a community health center in South Florida and women reported several important barriers that warrant attention. Given the high mortality rate of breast cancer among Black women, we have integrated a Patient Navigator in our health system to reduce barriers to annual breast cancer screening, follow-up care, and to facilitate timely access to treatment, thus ultimately reducing breast cancer health disparities and promoting health equity.

## Figures and Tables

**Table 1.** Sample Characteristics of Women (N=30)

Characteristics	n	Percent
**Age** (Years), Mean (SD)	54.3 ± 9.17	
**Race**		
Black/African American	30	100%
**Ethnicity**		
Hispanic/Latina	1	3.3%
Non-Hispanic/Latina	29	96.7%
**Country of Birth**		
United States	12	40%
Bahamas	1	3.3%
Barbados	1	3.3%
Brazil	1	3.3%
Haiti	11	36.7%
Jamaica	4	13.3%
**Education**		
Highschool or less	13	43.3%
Some college	8	26.7%
Graduated college	9	30%
**Annual Income**		
Less than $5,000	1	3.3%
$5,000- $9,999	4	13.3%
$10,000-$19,999	3	10.0%
$20,000-$29,999	4	13.3%
$30,000-$39,999	1	3.3%
$40,000-$59,000	2	6.7%
$60,000 or more	4	13.3%
Missing	11	36.7%
**Health Insurance**		
Medicaid	3	10.0%
Medicare	8	26.6%
Obama Care	6	20%
Private	5	16.7%
Uninsured	8	26.7%
**Employment**		
Working full-time	15	50.0%
Working part-time	3	10.0%
Unemployed	5	16.7%
Retired	2	6.7%
Student	1	3.3%
Disabled/Sick Leave	4	3.3%
**Family History of Breast Cancer**		
Yes	9	30.0%
No	21	70.0%
**Ever Been Diagnosed with Breast Cancer**		
Yes	1	3.3%
No	28	93.3%
Missing	1	3.3%
**How often do you do a breast self-exam (BSE)?**		
Monthly	8	47.0%
2 X Month	1	5.88%
2 X Year	2	11.76%
Weekly	1	5.88%
When I remember	2	11.76%
Never	3	17.65%
Missing	13	43.3%
**How often do you receive a mammogram?**		
Every 6 months	1	3.3%
Annual	16	53.3%
Every 2 years	4	13.3%
Every 3 years or more	4	13.3%
Never had a mammogram	5	16.7%

**Table 2-:** Perceived Barriers (N=30)

	Mean Score	Very Likely 5	Somewhat Likely 4	Neutral 3	Somewhat Unlikely 2	Very Unlikely 1
l.How likely is it that ‘Getting a mammogram would be **inconvenient for me’?**	2.033	7 (23.33%)	1 (3.33%)	-	-	22 (73.33%)
2. How likely is it that ‘Getting a mammogram **could cause breast cancer’?**	2.067	3 (10.0%)	-	9 (30.0%)	2 (6.67%)	16 (53.33%)
3.How likely is it that ‘The treatment I would get for breast cancer would be **worse than the cancer itself’?**	2.67	5 (16.67%)	3 (1.00%)	10 (33.33%)	1(3.33%)	11(36.67%)
4.How likely is it that ‘Being treated for breast cancer **would cause me a lot of problems’?**	3.10	11(36.67%)	2 (6.67%)	4 (13.33%)	1 (3.33%)	11(36.67%)
5.How likely is it that **‘Other health problems** would keep me from having a mammogram’?	2.20	7 (23.33%)	1 (3.33%)	2 (6.67%)	1 (3.33%)	19 (63.33%)
6.How likely is it that **‘My age** would keep me from having a mammogram’?	1.87	5 (16.67%)	-	-	2 (6.67%)	23 (76.67%)
7. How likely is it that ‘I would not get a mammogram because **my doctor already examines my breasts?’**	1.57	3 (10.00%)	1 (3.33%)	1 (3.33%)	-	25 (83.33%)
8.How likely is it that **‘Being afraid** of finding a breast lump **would keep me from having a mammogram’?**	1.60	2 (6.67%)	-	-	-	27 (90.00%)
9.How likely is it that **‘The trouble of having a mammogram** would keep me from getting one’?	1.20	1 (3.33%)	1 (3.33%)	-	-	27 (93.33%)
10.How likely is it that **‘Concern about pain with having a mammogram** would keep me from having one’?	1.93	5 (16.67%)	2 (6.67%)	1 (3.33%)	-	22 (73.33%)
11.How likely is it that **‘Being embarrassed about my body** would keep me from having a mammogram’?	1.53	4 (13.33%)	-	-	-	22 (86.67%)
12.How likely is it that **‘I will not have time to have a mammogram’?**	1.27	1 (3.33%)	1 (3.33%)	1 (3.33%)	1 (3.33%)	27 (90.00%)
13.How likely is it that **‘Not being able to afford a mammogram would keep me from having one’?**	2.53	10 (33.33%)	-	2 (6.67%)	-	18 (60.00%)
14.How likely is it that **‘Worrying about breast cancer** would keep me from having a mammogram’?	1.97	4 (13.33%)	1 (3.33%)	-	-	24 (80.00%)
15.How likely is it that **‘Concerns about being exposed to the x-ray** would keep me from having a mammogram’?	1.83	2 (6.67%)	2 (6.67%)	1 (3.33%)	-	25 (83.33%)
16.How likely is it that **‘I find it difficult to remember** to make an appointment for a mammogram’?	1.53	2 (6.67%)	2 (6.67%)	1 (3.33%)	-	25 (83.33%)
17.How likely is it that **‘Forgetting my appointment would keep me from getting a mammogram’?**	1.37	2 (6.67%)	-	3 (10.0%)	-	25 (83.33%)
18.How likely is it that **‘Being treated rudely at the mammogram centers** would keep me from having a mammogram’?	2.3	3 (10.0%)	1 (3.33%)	-	-	20 (66.67%)
19.How likely is it that **‘Not wanting to know would keep me from having a mammogram’?**	1.46	3 (10.0%)	-	1 (3.33%)	-	26 (86.67%)

**Table 3.** Mammography Screening Resources for Uninsured, Under-Insured, or Women with Limited Financial Resources

Local Organizations	Free Or Discounted	Websites
FoundCare Mammography Screening Center	Free mobile mammography service	https://foundcare.org/services/mammography-screening
Marie Louise Cancer Foundation	Free or discounted	http://mlcancerfoundation.org/home.html
Good Samaritan Hospital	Free or discounted	https://www.goodsamhosp.org/center-for-breast-health
Bethesda Women’s Health Center	Free based on income	https://www.bethesdacancercenter.org/womens-health-center
Northeast Health Center-Rivera Beach	Free or discounted	https://www.freemammograms.org/details/northeast-health-center-riviera-beach
Boca Regional Hospital MammoVan	Free or low-cost mammograms	https://www.brrh.com/Services/Lynn-Womens-Institute/Breast-Care/MammoVan/Mammovan-Appointment.aspx?furl=mammovan
Genesis Healthcare System	Free mammograms for those in need	https://www.genesishcs.org/news-search/free-mammograms-those-need-1
LIBBY’S LEGACY Breast Cancer Foundation	Free or discounted	https://libbyslegacy.org/mammogram-access-project-m-a-p/
**NATIONAL ORGANIZATIONS**		
Florida Breast and Cervical Cancer Early Detection Program (FBCCEDP)	**Free** (patients can go to various radiographic locations)	https://www.cdc.gov/cancer/nbccedp/success/mammograms-in-florida.htm
FREEMAMMAMOGRAMS.ORG	A list of locations offering free mammograms in florida	https://www.freemammograms.org/city/fl-west_palm_beach
